# Dietetic management of adults with Classical Galactosaemia in the UK: A care consensus document

**DOI:** 10.1016/j.ymgmr.2025.101277

**Published:** 2025-11-14

**Authors:** Louise Robertson, Simon Tapley, Alexa Sparks, Joy McDonald, Ellen Condie, Sarah Bailey

**Affiliations:** a University Hospitals Birmingham NHS Foundation Trust, Queen Elizabeth Hospital Birmingham, Mindelsohn Way, Edgbaston, Birmingham, West Midlands B15 2GW, UK; b Galactosaemia Support Group UK, 757 Manchester road, Stocksbridge, Sheffield, S36 1DQ, UK; c University Hospitals of Bristol and Weston NHS Foundation Trust, Bristol Royal Infirmary, Marlborough Street, Bristol BS1 3NU, UK; d The Newcastle Upon Tyne Hospitals NHS Foundation Trust, Royal Victoria Infirmary, Queen Victoria Road, Newcastle upon Tyne NE1 4LP, UK; e Belfast Health and Social Care Trust, Royal Victoria Hospital, 274 Grosvenor Road, Belfast BT12 6BA, UK; f University College London Hospitals NHS Foundation Trust, National Hospital for Neurology & Neurosurgery, Queen Square, London WC1N 3BG, UK; g All Wales Inherited Metabolic Disease Service, Cardiff and Vale UHB, University Hospital of Wales (UHW), Heath Park, Cardiff CF14 4XW, UK

**Keywords:** Galactosaemia, Adult Galactosaemia, Inherited metabolic disorders, Dietary management, Low galactose diet, Dietitian, Adult inherited metabolic disorders dietitian

## Abstract

Classical Galactosaemia is a rare, inherited metabolic disorder requiring lifelong dietary management. Despite increasing numbers of adults living with this condition, there is currently no formalised training or standardised guidance for United Kingdom (UK) dietitians managing adult patients. In response to this gap, a group of experienced UK dietitians specialising in inherited metabolic disorders collaboratively developed the first UK consensus document for the dietetic management of adults with Classical Galactosaemia. The guidance was formulated over a 12-month period through structured virtual meetings, with the objective of promoting consistency in clinical practice, supporting equitable service provision, and enhancing patient outcomes. Key areas addressed include dietary interventions, nutritional adequacy, bone health, dietary management with cognitive impairment, and reproductive health. The document also highlights critical gaps in the evidence base, particularly regarding individual galactose tolerance, the identification of sensitive biomarkers for monitoring, and the potential for dietary liberalisation in adulthood. This consensus guidance serves as a foundational resource for clinical practice and professional development, aiming to improve the quality and consistency of dietetic care for adults with Classical Galactosaemia across the UK.

## Highlights

• This is the first consensus document for the dietary management of adults with Galactosaeamia in the UK.
• The guidance covers dietetic assessment, interventions and follow up of adults with Galactosaemia.
• This guidance can be used as a tool for training dietitians new to the speciality, to help equity of care for patients with Galactosaemia in the UK and to support service provision.

## 1 Introduction

Galactosaemia is a rare, autosomal recessive, inborn error of carbohydrate metabolism which is caused by the deficiency of the enzyme galactose-1-phosphate uridyltransferase (GALT) [1]. The GALT enzyme is the second enzyme in the Leloir biochemical pathway which converts galactose into glucose. This leads to an accumulation of the metabolites galactose-1- phosphate (Gal-1-P), galactitol and galactonate [2]. This causes a life-threatening illness in neonates (liver failure, feeding difficulties, E Coli Sepsis and cataracts) and long-term complications in children and adults [3]. The primary management in the United Kingdom (UK) is a low galactose diet and this is recommended into adulthood [4]. The incidence of Galactosaemia in the UK is around 1 in 39,000 births [5] higher in Ireland with 1 in 16,000 births [6]. Despite good adherence to the low galactose diet, long term complications can occur including cognitive deficits, speech and language difficulties, neurological abnormalities and primary ovarian insufficiency in females [3]. The cause of these problems is unknown but may result from release of free galactose from endogenous sources such as turnover of glycoproteins and glycolipids that are released into the plasma [7]. The amount of endogenous galactose released is constant and is not influenced by diet [8]. Adults have been shown to have slower rates of release of endogenous galactose than newborns [9].

We have known about Galactosaemia for over 100 years, with the first description of an infant with Galactosaemia being reported in 1908 and the first report of successfully using a milk-free diet to treat Galactosameia in 1935 [10]. Even though we have known about the low galactose diet for the management of Galactosaemia for many years, we have little evidence if the diet can be relaxed in adulthood. The International Clinical guidelines for Galacotsaemia [4] now advise that the diet should only be a restriction of galactose from milk and milk products.

Adults with Galactosaemia are living to older age, but often still rely on parents, relatives or carers for support and many suffer from cognitive impairment [3,11]; management and follow up in adulthood is important.

As the primary management for Galactosaemia is a low galactose diet, dietitians are integral to patient care and are often the first point of call for patients and their families. People with Galactosaemia should be followed up throughout life in specialist inherited metabolic disorder (IMD) centres and transitioned to specialist IMD adult services [12]. There is no specific training for dietitians specialising in adults with Galactosaemia in the UK and training is often ‘on the job’ with support from other dietitians and health care professionals in the field. Management is guided by the International Clinical Guidelines for the Management of Galactosaemia [4]. Only four of these forty recommendations are related to dietary management, giving only a small amount of detail. The aims of this care consensus document are to agree a standard of dietetic care for adults with Galactosaemia in the UK, to outline service provision required for optimal dietetic care, to outline the role of the adult IMD dietitian and to promote equity of patient care throughout the UK.

## 2 Materials and methods

Six UK dietitians (including England, Wales and Northern Ireland) specialising in adults with inherited metabolic disorders met virtually over a year (2022−2023) to write and discuss the guidance until 100 % agreement was reached. The structure of the guidance was based on the ‘The Dietetic Management of Adults with Phenylketonuria (PKU) in the UK: A Care Consensus Document’ published in 2022 [13]. The guidance was written based on ‘The International clinical guidelines for the management of Classical Galactosaemia: diagnosis, treatment and follow up’ [4], evidence and clinical experience.

Once written, the guidance was reviewed by the British Inherited Metabolic Disorders Group (BIMDG) dietitians' subgroup, the BIMDG committee, Galactosaemia Support Group (UK) committee and four adults with Galactosaemia. All stakeholders were happy with the guidance and small improvements were suggested and incorporated.

The following areas were discussed; The scope of the guidance, aims and objective of the guidance, the duties of the adult IMD dietitian, the policy delivery and implementation, dietetic assessment and interventions for adults with Galactosaemia and monitoring and assurance.

## 3 Results

The full guidance can be found in appendix A. The guidance addresses the standards of dietetic care and interventions for adults with Galactosaemia. The aspects of care in the guidance include the following:

• Aims of dietetic care
• Dietetic assessment
• Interventions which include:
  o Low galactose diet
  o Allowance of low galactose cheese
  o Allowance of butter oil and ghee
  o Allowance of caseinates
  o Allowance of moderate amounts of offal, soy sauce and fermented soy products
  o Guidance on suitable alcohol
  o Guidance on adequate calcium / vitamin D / iodine intake
  o Guidance on galactose content of medications
  o Bone health
  o Females and pregnancy
  o Adults who have relaxed their low galactose diet
  o Patients with learning difficulties
  o Hospital admissions
  o Monitoring
  o Nutritional blood tests
  o Advances in research
• Follow up
  o Follow up appointments
  o Follow up contact in between appointments
  o Guiding patient to access support from other agencies or other health care professionals
  o Discharge / transfer arrangements if appropriate
• Potential outcome measures
• Resources

It is recommended that this care consensus document is reviewed every 3 years or is updated within 6 months if any new evidence or guidance is published that necessitates a change in practice. The authors also recommend that all services should perform an audit by using a representative sample of patients, using this guidance as a benchmark.

## 4 Discussion

This is the first dietetic consensus guidance for the dietary management of adults with Galactosaemia in the UK. It is a practical interpretation of the International Clinical Guidelines for the management of Classical Galactosaemia [4] but also includes evidence and the authors' collective clinical experience. It should be used alongside the clinical judgment of the dietitian to tailor their advice to provide patient centred care. It can be used as a tool for training dietitians new to the speciality, to help equity of care for patients with Galactosaemia in the UK and to support service provision. The guidance examines different aspects of care in relation to adults with Galactosaemia. The below issues are pertinent to the dietary care of adults with Galactosaemia.

### 4.1 Quantity of galactose in the diet

After the initial crisis in newborns with Galactosaemia and the removal of galactose from the diet, there is limited evidence to stay how strict the diet should be in childhood and then into adulthood. There is increasing evidence that the diet should only be a restriction of galactose from dairy products. The GalNet registry (509 Classical Galactosaemia patients) [3] found that patients with a strict diet (lactose restriction and restriction in fruits and vegetables) developed neurological complications more frequently than patients on a less strict diet (just a restriction of galactose from dairy products). Other studies have found no difference in the rigour of diet on long term outcomes [11,14]. There are short term small studies looking at the effect of increased galactose intake in adults [[15], [16], [17]]. One issue when looking to relax the diet is how to monitor the effects of increased galactose intake. Gal-1-p is used to monitor dietary adherence, especially in children but it is not thought to be a good marker of adherence in adults [15,16]. IgN-glycan profiling has been investigated and may be a more sensitive marker [15] and could be used to determine optimum personalised glycosylation profiles and therefore different dietary galactose intakes for each person with Galactosaemia [17]. In a small study of 10 patients, favourable N-Glycan profiles were shown at different levels of dietary galactose intake for each patient. One patient showed an unfavourable profile at 1000 mg, whereas another had a favourable profile at 4000 mg of galactose, showing galactose tolerance may be individual [15]. In a larger study with 95 Classical Galactosaemia patients from 5 centres in 4 countries, patients were grouped by approximate daily galactose intake (<200 mg, 200-500 mg and 501-1000 mg) and looked at N-glycan profiling [17]. They showed a decrease in galactosylation and sialylation and an increase in fucosylation in Classical Galactosaemia compared to controls. Galactosylation, sialylation and fucosylation play a role in the function and stability of proteins and lipids. For patients who were homozygous for p.Gln188Arg (severe phenotype) (*n* = 50) they found a higher IQ (*P* < 0.005) in the 501-1000 mg galactose intake group compared to the <200 mg group. They also found improvements in monosialylated, monogalactosylated, and monoantennary glycans with increase galactose intake. To put this into perspective, 100mls of cow's milk equates to 2400 mg of galactose, so 1000 mg galactose is no more than 50mls of milk per day or allowing patients to use small amounts of butter, cheese with slightly higher galactose content or having baked goods with added milk such as bread, biscuits or cakes [18]. More work is needed in this area before advice can be changed.

There are reports in the literature of patients being diagnosed later in life for reasons relating to the absence of newborn screening or absence of acute neonatal symptoms or false negative screening. Quelhas et al., (2024) present three cases where lack of treatment has caused significant consequences beyond the usual development and progression of Primary Ovarian Insufficiency (POI) and neurological symptoms [19]. However, there are also examples of patients missing diagnosis in childhood and managing relatively well into adulthood [20,21].

There are then examples of patients where galactose restriction has been completely lifted at an early age. These two adult patients had Galactose intakes of 2690 mg/ day from a three-day diet record [22] and 9000 mg/ day [23]. Both had symptoms not dissimilar from the course of development in those patients who were treated with lifelong dietary restriction.

Together these cases reinforce the theory that there may be differing tolerance of galactose in different phenotypes. However, without specific biomarkers, defining this tolerance would incur significant risk.

### 4.2 Cataracts and diet rigour

Patients with Galactosaemia are at increased risk of developing cataracts. Cataracts form in Galactosaemia through the accumulation of galactitol in the lens. Galactitol is not only osmotically active but also tends to accumulate in the lens fibre cells, due to the high concentration of aldolase reductase at the anterior side of the lens. This accumulation can lead to lens swelling, cell lysis, and ultimately cataract formation [24].

Widiger et al., (2010) [25] retrospectively reviewed the incidence and severity of cataracts in patients attending their centre with Classic Galactosaemia and identified those with cataracts diagnosed by an ophthalmologist. Compliance to diet was also reviewed and compared with a matched control group. Of 100 active patient charts, 14 had cataracts, six of these persisted whereas eight regressed. Three occurred soon after birth. Age at cataract formation varied from soon after birth to 19 years of age. There was no significant difference in the cataract group between those who were compliant and those who were noncompliant with diet (*p* = 0.09). There was no difference in compliance between the cataract group and the control group (*p* = 0.16). None of the cataracts found were affecting vision. A direct relationship between dietary compliance and cataract formation was not demonstrated.

Garrett et al., (2024) found the prevalence of cataracts in adults (30–79 year olds) with Galactosaemia did not differ significantly between cases and controls (32.78 % vs. 18.42 %; *p* = 0.101). The authors suggest this may be because cataracts are more common in older adults in the general population [11].

### 4.3 Bone health and nutrient intake

In their systematic review and meta-analysis Van Erven et al. (2017) found that bone health is affected in patients with Classical Galactosaemia and they are at higher risk of low bone mass than the general population [26]. International Galactosaemia guidelines [4] recommend bone mineral density (BMD) screening and any indicated treatment, as well as annual dietary assessment of calcium and vitamin D intake including serum measurements. Recommended daily allowances (RDAs) for calcium, magnesium, vitamin D and iodine should be met. Regular exercise, including muscle strengthening, should also be recommended [27], as well as smoking cessation and limiting alcohol intake to no more than 14 units per week. A unit of alcohol equates to 10 ml or 8 g of pure alcohol. For example, one single shot (25mls) of spirits equates to 1 unit, a small (125mls) glass of wine is 1.5 units and a pint of lower strength beer/larger/cider equates to 2.4 units [28].

Avoiding dairy (animal milk and milk products), as per the recommendation for Classical Galactosaemia, can result in inadequate calcium intake [29]. A good dietary source of calcium should contain adequate quantities and be sufficiently bioavailable: lower levels of absorbable calcium in non-dairy foods can limit contributions to bodily calcium requirements [30,31]. Hence, dietary advice should be tailored to consider bioavailability in non-dairy foods, and calcium-fortified foods and drinks advised, alongside supplementation as needed.

Iodine deficiency can have many clinical implications including hypothyroidism, goitre and impaired cognitive function. Milner et al., (2023) indicated an increased risk of iodine insufficiency among children (1–18 years) with Classical Galactosaemia in Ireland, compared to the general population [31]. Iodine intake should be assessed, and fortified alternative milks, permitted cheeses, white fish and eggs encouraged, with supplements considered.

There is a widely held consensus that it is possible to meet dietary requirements with diets that exclude dairy milk and milk products [32]. However, this is not always feasible in practice. Supplementation of diet with combined calcium and vitamin D has been shown to reduce fracture risk in the general population [33]. There has been some concern about the cardiovascular risks associated with calcium supplementation [34]. In a narrative review of the evidence regarding cardiovascular risk Zarzour et al., (2023), conclude the bulk of the evidence does not support this; that calcium and vitamin D should be preferably obtained from diet, with supplements useful in patients unable to achieve adequate intake from dietary sources [35]. The Royal Osteoporosis Society has provided practical clinical guidance on when and how to supplement vitamin D and calcium [36].

### 4.4 Suitable cheese in Galactosaemia

A good source of dietary calcium and iodine is cheese. Since 2000 the Galactosaemia Support Group in the UK have tested over 190 different samples of cheese looking at their lactose and galactose content [37,38] unpublished data). The International guidelines state that a cheese is suitable to eat if the galactose content is less than 25 mg/100 g. The UK allows Extra Mature Cheddar, Vintage Cheddar, Italian Parmesan, Grana Padano, Emmental, Gruyere, Comte, Jarlsberg, Original Babybel (red) and more recently low fat extra mature cheddar and Pecorino (unpublished data). Other countries have done similar testing and also allow some hard cheeses [18,39,40]. Encouraging adults to try and include some cheese in their diet will help to increase their dietary calcium and iodine intake. In practice we often see that adults with Galactosaemia are not keen to add new foods into their diets so starting to introduce allowed cheese during weaning and childhood can help develop their preference for cheese.

### 4.5 Fermented soy foods

The fermentation process of the soybeans releases free galactose from oligsaccarides. The free galactose of miso paste, soy sauce and sufu was measured by Van Calar et al., (2014) and found to be between 290 and 912 mg per 100 g [41]. Although high, these foods are often used as condiments or flavourings so in minimal amounts. Therefore, the International clinical [4] guideline recommended that all fermented soy-based products e.g. miso, tempeh, natto and sufu can be allowed in the diet in moderation.

### 4.6 Alcohol

Any alcohol containing dairy products e.g. cream liqueurs should be avoided. Some beers can have lactose added to produce a sweeter taste. These include New England IPAs (NEIPA), modern sour beers (usually with fruit), milkshake IPAs and dessert stouts. Lactose can also be added to low alcoholic beers to give extra body. Lactose content varies but can range from 2 to 5 % of the beer, meaning a typical 440 ml tin of beer could contain 4400-11,000 mg galactose. Allergen labelling applies to alcoholic drinks so it must declare on the beer packaging if it contains lactose [42]. All other types of alcohol including wine with added caseinates are acceptable to have within safe alcohol limits [28], as the galactose content is very low.

### 4.7 Learning difficulties

One of the issues with educating adults with Galactosaemia is the wide spectrum of cognitive abilities. Some adults with Galactosaemia live independently while others still live with their parents or live in care homes. The GalNet registry [3] reported on 509 people with Galactosaemia, 276 who were over 18 years. 85 % of patients were reported to have brain impairments and 52 % global development delay. Garrett et al. (2024), reported on a survey to 92 adults with Galactosaemia from Europe and North America. They compared controls (*n* = 38) and adults with Galactosaemia (*n* = 92) and found that 8.33 % of controls reported problems with cognitive function compared to 75.8 % of the Galactosaemia adults. They found more of the Galactosasmia adults were living in assisted living or with parents or relatives than controls (41.8 vs 5.3 %) [11]. Many adults with Galactosameia rely on parents or carers to bring them to appointments and to help understand and follow the dietary advice [43,44]. Dietary education and resources need to be easy to understand with simple messages. The education often needs to be directed towards carers or care home staff as well as the patient.

### 4.8 Primary ovarian insufficiency and pregnancy

Primary ovarian insufficiency (POI) is a common long-term complication in females with Galactosaemia [3,11,45,46] and it is thought to be independent of the strictness of the low galactose diet [46]. Several studies have found that not all females with Galactosaemia try to conceive [11,47] but of those that did there was a higher-than-expected pregnancy success. Van Erven et al. 2017, found that 30 % of women with Classical Galactoseamia and POI who actively attempted to conceive succeeded within one year and this increased to 50 % within 2 years [47]. Garrett et al., (2024) reported that all the women that attempted pregnancy had spontaneous menarche [11]. Normal pregnancies with healthy babies have been reported in women with Classical Galactosaemia while following a low galactose diet [[48], [49], [50]] and safe breastfeeding (while following a low galactose diet) has been reported by Galactosaemic mothers to their non Galactosaemic babies [50]. If women are trying to conceive or become pregnant then is it is important they are following a nutritionally adequate diet for pre-conception and pregnancy while following their usual low galactose diet.

### 4.9 Clinic attendance in adulthood

Galactosaemia is a long-term condition which should have lifelong treatment and metabolic-specialist follow-up [12]. Maintaining engagement and clinic attendance into adulthood is important. As has been discussed, clinical monitoring, education on advances in research and developments in dietary advice and intervening to optimise bone health and support with POI and pregnancy are vital elements of care. Delivering this requires regular contact with patients and carers, where options of video or telephone appointments could be utilised at intervals, along with encouraging a patient-led approach to seek support and information as needed. Engagement also allows for clinicians to support in advocating for patients in the workplace and with applications for financial support, for example.

As adults with Galactosemia are known to have a high instance of cognitive impairment [11] it is possible that this can impact consistent clinic attendance, ability to understand advice and engage with support. Randall et al. (2022,2023) observed that many adults with Galactosaemia rely on support from parents and carers at appointments, which should be encouraged [43,44]. Additionally, methods such as text reminders and easy read communication and resources should be employed to support independent access.

## 5 Conclusion

This document represents the first consensus dietetic guidance for adults with Galactosaemia in the UK. It defines the essential role of the dietetic team in managing adult patients and aims to support consistent service delivery and equitable care across the country. As the Galactosaemia population ages, there is a growing need for outcome-based research to inform future care strategies. Key areas for future investigation include understanding individual galactose tolerance, identifying sensitive biomarkers to monitor metabolic control, and determining whether and when dietary restrictions can be safely relaxed.

Engaging patients in ongoing care and keeping them informed of emerging evidence and treatment options, such as potential new medications, will be vital. This evolving landscape underscores the importance of continued collaboration, research, and innovation to improve long-term outcomes for individuals living with Galactosaemia.

## CRediT authorship contribution statement

**Louise Robertson:** Writing – review & editing, Writing – original draft, Project administration, Methodology, Funding acquisition, Conceptualization. **Simon Tapley:** Writing – review & editing, Writing – original draft, Methodology, Conceptualization. **Alexa Sparks:** Writing – review & editing, Writing – original draft, Methodology, Conceptualization. **Joy McDonald:** Writing – review & editing, Writing – original draft, Methodology, Conceptualization. **Ellen Condie:** Writing – review & editing, Methodology, Conceptualization. **Sarah Bailey:** Writing – review & editing, Methodology, Conceptualization.

## Funding

This research did not receive any specific grant from funding agencies in the public, commercial, or not-for profit sectors for writing the consensus guidance and manuscript. Funding was acquired for publication costs from the Galactosaemia Support Group UK and the University Hospitals Birmingham Charity (IMD).

## Declaration of competing interest

No conflict of interests.

## Figures and Tables

**Table:** 

1	Glossary
2	Scope
3	Introduction to the clinical guideline
4	Aims and objectives of this guideline
5	Duties
6	Guideline Policy delivery and implementation
6.1	Key stakeholders
6.2	Dietetic assessment and interventions for adults with Galactosaemia
7	Monitoring and assurance
8	References

**Table:** 

Activity	Information
Aims of dietetic care	• To support adults with Galactosaemia to identify their personal aims and goals through the life cycle. • To ensure the patient is fully informed on best practice management of Galactosaemia in accordance with International clinical guideline for the management of Classical galactosemia [4] and to support the patient to make informed treatment decisions. • To encourage life-long, galactose-restricted diet that only eliminates sources of lactose and galactose from dairy products. • To encourage dietary variety and avoid exclusion of allowed foods. • To ensure calcium intake is sufficient for age and stage of life in accordance with dietary guidelines for the general population, focusing first on food sources and supplementing with calcium supplements if required. • To ensure regular monitoring of plasma total 25-OH-vitamin D levels and supplementation in line with recommendations for the general population. • To assess the diet for nutritional adequacy, with particular attention to nutrients required for bone health (e.g., Vitamin D & Iodine). • To follow the guidance of specialists in the management of osteoporosis or osteopenia and avoid over treatment of calcium supplementation. • To promote regular exercise and weight bearing activities for bone health. • To help promote a healthy weight.


6.2.1 Dietetic Assessment	

For all patients review:	• Confirm identification of the patient and seek consent for assessment. • Medical/surgical history. • Environmental factors. • Psychosocial considerations e.g., change of living circumstances. • Anthropometry: weight, height, body mass index (BMI). • Current clinical issues. • Relevant medications including calcium / vitamin D / multivitamin supplements. Consider lactose content of medications. • Biochemistry: nutritional bloods, including annual plasma total 25OH Vitamin D. • Diet history to include assessment of: • any dietary sources of galactose from dairy products • overall dietary adequacy, including assessment of calcium, vitamin D and iodine intake • use of non-dairy alternatives (e.g., plant-based milks, yoghurt, cheese, cream etc.) and whether these are fortified with calcium, vitamin D and iodine • intake of allowed, minimal galactose, calcium rich foods (e.g., allowed cheese) and confidence with incorporating these into the diet • unnecessary restriction of allowed foods / food groups and rational. • menu planning and cooking skills. • history of nutritional deficiencies. • Vitamin / mineral / trace element supplementation, including calcium, vitamin D and iodine. Consider prescribed versus actual intake. • Confidence in reading food labels and choosing suitable low galactose alternatives. • Identify any barriers to adherence of treatment related to Galactosaemia and any specific concerns the patient has relating to their Galactosaemia. • Relationships with food if concerns are raised. • Membership / awareness of support groups (Galactosaemia Support Group, UK). • Exercise / activity behaviours including weight bearing exercise. • Date, result and treatment recommendations of last Dual-energy X-ray absorptiometry (DEXA) scan.


6.2.2 Interventions	

Low galactose diet	• Advise on a low galactose diet that only eliminates galactose from all dairy (animal) milks but permits galactose from non-milk sources (e.g., vegetables, fruit, pulses, soya beans, non-fermented soy-based products). • Advise on a low galactose diet for life (as inadequate evidence for an age-related recommendation). • Educate on label reading and how to look for ‘milk’ on labels. This should be in bold due to allergy legislation. • Educate that allergen / milk cross contamination is not a problem in a diet for Galactosaemia. • Educate that lactofree / lactose free foods are not suitable as they still contain galactose. • Occasional, accidental galactose intake will not cause acute serious problems.

Allow Cheese with a very low galactose content	• Cheese with a galactose content of <25 mg/100 g is allowed in the diet. In the UK, cheese that has been tested and allowed includes UK extra mature cheddar, UK low fat extra mature cheddar, UK vintage cheddar, Italian Parmesan, Grana Padano, Pecorino, Emmental, Gruyere, Comte, Jarlsberg and Babybel TM original [4,37,38]

Allow Butter oil and Ghee	• Butter oil (butter fat), anhydrous milk fat (milk fat) and ghee have negligible galactose content, and these ingredients can be eaten within foods providing there is no other source of milk in the food [4,51]

Allow caseinates	• Sodium caseinate and calcium caseinate (food additives) are allowed in the diet as they are used in small amounts in manufactured foods [4,41]

Allow moderate amounts of offal, soy sauce and fermented soy products	• The galactose content of offal is unknown, but there is no direct evidence of harm. Therefore, these products can be eaten in moderate amounts [4] • Fermented soy products are allowed in small quantities that are typically used in the diet. They include miso, tempeh, natto and sufu [4,41]

Alcohol	• Wine with added caseinates is acceptable to have (within UK alcohol intake guidance [28]) as the galactose content is very low. • Avoid craft beers with added lactose and alcoholic drinks containing dairy products e.g. cream liqueur.

Adequate Calcium intake	• Ensure patient is meeting calcium recommendations for age [52] • Encourage allowed cheese as it is a good source of calcium. • Ensure milk alternatives are fortified with calcium. • Calcium is efficiently absorbed in smaller doses; high dose calcium supplements (>1000 mg) should be spilt into two doses. Avoid over supplementation.

Adequate vitamin D intake	• Encourage foods containing vitamin D e.g., fortified milk alternatives, oily fish, meat, and eggs. • Vitamin D should be supplemented. • Reference nutrients intake (RNI) is 10μg daily in adults in the UK [53] • Can be combined with a calcium tablet or on its own.

Iodine	• Encourage sources of iodine e.g. white fish, allowed cheese, eggs. • Encourage milk alternatives fortified with iodine [31]. • If unable to meet iodine requirements [54], consider an iodine supplement (multivitamin).

Medication	• Some medications may use lactose as a filling or bulking agent. • For adults the quantity may be very small and be safe to have. • Discuss with medical team the risks vs the benefits of taking the medication. • If buying over the counter preparations encourage patient to check ingredients with the pharmacist.

Bone Health	• Discuss with patients that they are at risk of low bone mineral density due to a restricted diet and having galactosaemia. • Ensure a healthy diet including adequate calcium and vitamin D. • Encourage regular exercise, including muscle strengthening [27]. • Encourage to stop smoking and limit alcohol intake to no more than 14 units per week [28]. Signpost to smoking cessation support services as appropriate. • Dual energy X-ray absorptiometry (DEXA) scans every 5 years to monitor bone density.

Females	• Over 80 % of females with Classical Galactosaemia develop primary ovarian insufficiency (POI). • The clinical spectrum varies widely. • Females with POI may be advised by their medical team to take hormone replacement therapy (HRT). • Females are at higher risk of low bone mineral density due to primary ovarian insufficiency. • Encourage regular exercise alongside a healthy diet that includes plenty of calcium, fruit, vegetables, wholegrains to keep bones healthy and protect against heart disease.

Pregnancy	• Chances of pregnancy are reduced due to POI, but pregnancies have been reported [45]. • Advise to follow usual low galactose diet during pregnancy. • Folic acid to be taken in line with government recommendations. • Ensure meeting calcium, vitamin D and iodine requirements. • Breastfeeding while following a low galactose diet has been found to be safe [50].

Adults who have relaxed their low galactose diet or do not follow a low galactose diet	• Discuss current guidance (guidelines) which recommends a lifelong low galactose diet. • If the patient is making an informed decision about galactose intake and does not want to change, then discuss with team, document and support decision. • Encourage a healthy, balanced diet including adequate intake of calcium, vitamin D and iodine.

Patients with learning difficulties	• To work with care staff / family members to support adherence to treatment. • Provide teaching and resources to care staff and family members as required.

Hospital admissions	• A low galactose diet should be followed if admitted to hospital. • To liaise with local hospital dietitians. • If an enteral tube feed or oral nutrition supplement is needed, choose a minimal galactose product.

Monitoring	• Red blood cell Gal-1-P and urinary galactitol have limited use as markers of adherence to the low galactose diet in adults. • Diet histories / recalls / food frequencies / questionnaires can be used to monitor galactose intake, adequate calcium, vitamin D and iodine intake and known/accidental consumption of lactose/galactose. • DEXA scans – 5 yearly.

Nutritional bloods	• To include annual plasma total 25-OH-vitamin D.

Advances in research	• To discuss new research, treatment or guidelines as appropriate.


6.2.3 Follow up	

Follow Up Appointment	• Agree follow up with patient. • Maximum of 12-month intervals. • The option of video or telephone appointment to be considered if appropriate. • Encourage face to face appointment at least every 2 years.

Follow-up contact in between clinic appointments	• Provide contact details for queries or help between appointments. • Encourage patient-led approach to seek support and information as required. • Update patients on significant developments in dietary treatment, for example development in suitable foods available. • Update patients on significant resources made available.

Guiding patient to access support from other agencies or other HCP	• Refer to other health care professionals as needed, e.g., learning disability team, psychology, speech and language therapy. • Signpost to services outside of the NHS as appropriate. • Provide supporting letters as required, e.g., for travel, applying for benefits (for example personal independent payment), employers.

Discharge / transfer arrangements if appropriate	• Discharge arrangements will vary between services. • Galactosaemia is a long-term condition which should have lifelong treatment and metabolic-specialist follow-up. • Provide dietetic handover to another centre if patient relocates, e.g., university students, moving house.

6.2.4 Potential outcome measures	• Patient reported experience measures (PREMs) and patient reported outcome measures (PROMs). • Knowledge and skills of managing diet. • Attendance of appointments. • Adherence to vitamin and mineral supplements as required. • Variety and nutritional adequacy of diet. • Body Mass Index. • No biochemical evidence of nutritional deficiencies.

6.2.5 Resources	• GSG dietary information including – Diet for Galactosaemia in adults, Calcium, vitamin D and bone health in Galactosaemia. • GSG website information including label reading and picture diet booklet. • Relevant centre resources.


## Data Availability

This is a review and no data used.

## Appendix A Guidance for the dietetic management of adults with Classical Galactosaemia in the UK

**CATEGORY:** Guideline.

**CLASSIFICATION:** Clinical.

**PURPOSE:** This guideline outlines the Dietitian care pathway for adult patients (16+ years) with Galactosaemia under the care of Adult Inherited Metabolic Disease (AIMD) teams in the UK.

**Version Number:** 1.0.

**Owners:** BIMDG Dietitians Adult Group.

**Authors (Working party):** Louise Robertson (University Hospitals Birmingham NHS Foundation Trust + Galactosaemia Support Group UK), Sarah Bailey (All Wales Service, Cardiff and Vale UHB), Ellen Condie (University College London Hospital), Joy McDonald (Belfast Health and Social Care Trust), Alexa Sparks (The Newcastle Upon Tyne Hospitals NHS Foundation Trust), Simon Tapley (University Hospitals of Bristol and Weston NHS Foundation Trust).

**Review Date:** 3 years.

**Essential Reading for:** Dietitians working with adult Galactosaemia patients.

**Information for:** Members of the MDT working with patients with Galactosaemia.

### Contents

1. Glossary

• Patient: patient or patient advocate.
• Adult Inherited Metabolic Diseases Dietitian (AIMD dietitian): a dietitian who works with adults who have an inherited metabolic disease (IMD) and has been trained in IMD.
• Patient centred approach: treating the patient as an individual and an equal partner in their healthcare management.
• Psychosocial: how social conditions affect mental health or how someone copes with Galactosaemia.
• GSG: The Galactosaemia Support Group – patient support group in the UK.
• BIMDG: The British Inherited Metabolic Diseases Group - Health professionals interest group in the UK.
• Dairy and Dairy products: all milk that is derived from an animal e.g., cow, sheep, goat and its products e.g., cheese, butter, yoghurt, cream.

2. Scope

• This guideline outlines the dietetic care pathway for adult patients (16+ years) with Galactosaemia under the care of Adult Inherited Metabolic Disease (AIMD) teams in the UK.
• The role of the AIMD dietitian in Galactosaemia care is highlighted.
• This document is to be used alongside the clinical judgment of the dietitian to tailor care to the adult with Galactosaemia.
• The roles of other healthcare professionals are noted, although the entirety of their role in the pathway has not been included.

3. Introduction to the clinical guideline

• Classical Galactosaemia (type 1) is a rare inborn error of carbohydrate metabolism which is caused by the deficiency of the enzyme galactose-1-phosphate uridyltransferase (GALT). The GALT enzyme converts galactose into glucose. Deficiency of GALT leads to an accumulation of the metabolites galactose-1- phosphate (Gal-1-P), galactitol and galactonate [4]. This causes a life-threatening illness in neonates and long-term complications in children and adults [4].
• The incidence of Galactosaemia varies worldwide. It has a prevalence in western countries of between 16,000 and 60,000 live births [3]. In the UK, the incidence is about 1 in 39,000 births [5] and 1:16,000 in Ireland [6].
• The primary management is a low galactose diet and The International Clinical Guidelines for Galactosaemia advocate a low galactose diet for life [4].
• Across the UK there are recognised adult IMD centres.
• This guideline is necessary to achieve the following:
  ▪ Standardise the dietetic care of all adult patients with Galactosaemia across the UK.
  ▪ Provide a framework to support the dietitian's decision-making around treatment of adult patients with Galactosaemia.
  ▪ Assist development and supervision of AIMD dietitians in the UK and to support equitable standards of care to adults with Galactosaemia.

4. Aim and objectives of this guideline

• To outline the role of the AIMD dietetic team in provision of care to adults with Galactosaemia.
• To promote equity of patient care throughout the UK.
• To agree standards for patient care to ensure patient safety and optimal care provision, referring to International clinical guidelines for the management of Classical Galactosaemia: diagnosis, treatment and follow up, 2017 [4].
• To outline the service provision required to provide optimal dietetic care.

5. Duties

• It is the responsibility of the AIMD dietetic service to implement the procedures and provide best practice care as outlined in this document.
• All members of the AIMD dietetic team have a role in advocating for adults with Galactosaemia to receive the care that is aligned with this document, unless this is otherwise indicated during the review.
• All members of the AIMD dietetic team are required to escalate any aspects of patient management of concern not within the dietetic scope of practice to an appropriate IMD team member.

6. Policy delivery and implementation
  6.1. Key stakeholders in the guideline
  • The AIMD Dietitians in AIMD centres across the UK.
  • The IMD teams including consultants, clinical nurse specialists, and dietetic assistants to support implementation of the guideline.
  • Patients, their families, carers and advocates.
  • The British Inherited Metabolic Diseases Group (BIMDG).
  • Galactosaemia Support Group (GSG).
  6.2. Dietetic assessment and interventions for adults with Galactosaemia

7. Monitoring and assurance

Guideline group: The guideline working group will review this document every three years to ensure it remains up to date and informed by clinical guidance and evidence. The guideline was written in December 2024, and the review date will be in December 2027. If any new evidence or guidance is published which requires a change in practice, it will be updated within 6 months of publication. The working group will meet to update this.

Service level: AIMD dietetic services should use this SOP as a benchmark to audit provision of services to patients, to highlight gaps in services, and to identify changes in service provision required to conform to the latest guidelines and requirements and the in development of business cases.

It is recommended that each AIMD dietetic service complete an annual audit on a representative sample of patients on key outcomes outlined in this document (such as frequency of consultations) and act on the findings of the audit appropriately.
